# *De Novo* Assembly and Annotation of *Salvia splendens* Transcriptome Using the Illumina Platform

**DOI:** 10.1371/journal.pone.0087693

**Published:** 2014-03-12

**Authors:** Xiuxiu Ge, Hongwei Chen, Hongli Wang, Aiping Shi, Kefeng Liu

**Affiliations:** 1 College of Biological Science and Engineering, Beijing University of Agriculture, Beijing, P. R. China; 2 College of Urban & Rural Development, Beijing University of Agriculture, Beijing, P. R. China; 3 College of Horticulture, Beijing University of Agriculture, Beijing, P. R. China; East Carolina University, United States of America

## Abstract

**Background:**

As an important perennial herbaceous flower, *Salvia splendens* possesses high ornamental value. Understanding its branching processes may help scientists select the best plant type. Although *Salvia splendens* is a frequently-used horticultural flower, only limited transcriptomic or genomic research is available in public databases. In the present study, we, for the first time, constructed a comprehensive dataset for *Salvia splendens* through *de novo* high-throughput transcriptome sequencing.

**Methodology/Principal Findings:**

We performed *de novo* transcriptome sequencing on two different branching type plants (Strain 35 and Cailinghong) using the Illumina paired-end sequencing technology. For Strain 35, a total of 16,488,829 reads were generated and assembled into 38,498 unigenes, with a mean length of approximately 779 bp. For Cailinghong, 16,464,713 reads were generated and assembled into 34,302 unigenes, with a mean length of approximately 812 bp. Moreover, a total of 49,310 unigenes for *Salvia splendens* were identified, among them 33,925 (68.80%) were annotated in the non-redundant NCBI database, 25,371 (51.45%) were annotated in the Swiss-Prot database, while 24,888 (50.47%) and 9,896 (20.07%) unigenes were assigned to gene ontology categories and clusters of orthologous groups, respectively. Using the Kyoto Encyclopedia of Genes and Genomes pathway database, we identified 134 differently expressed unigenes between Strain 35 and Cailinghong, and then these unigenes were mapped to 79 pathways. In addition, we detected 2,453 simple sequence repeats (SSRs).

**Conclusions:**

We obtained a comprehensive transcriptomic information from this work and provided a valuable resource of transcript sequences of *Salvia splendens* in public databases. Moreover, some candidate genes potentially involved in branching were identified. Furthermore, numerous obtained SSRs might contribute to marker-assisted selection. These data could be further utilized in functional genomics studies on *Salvia splendens*.

## Introduction

*Salvia splendens* belongs to *Salvia*, which is an important herbaceous flower used in the configuration of parterre. Flowers of *Salvia splendens* can be used as a source of food pigment. Despite its significant economic contribution, there are few studies on the genetic or genomic of *Salvia splendens*, and until December 2012, only 33 gene sequences were available in NCBI database. Moreover, studies on these genes have mainly focused on anthocyanin metabolic pathways.

To increase branches and corresponding flowers, it is necessary to manually pinch at least twice during the cultivation of *Salvia splendens*. Our newly-developed variety Cailinghong (Variety number: Jing S-SV-SS-002-2010) is a plant-type mutant derived from normal Strain 35. Cailinghong has a strong branching ability; therefore, it can grow into spherical plant-type naturally without pinching, saving the manpower in the factory production for *Salvia splendens*. Signaling pathways involved with branching in *Salvia splendens* can be studied through the global analysis of the differentially expressed transcripts between Strain 35 and Cailinghong.

The emergence of the next generation sequencing (NGS) technology makes the rapid genome sequencing become possible. RNA sequencing has advantages compared with the whole genome sequencing because only transcribed regions of the genome are analyzed [1]–[4]. Moreover, RNA sequencing can provide abundant information on gene expression, gene regulation and amino acid content of proteins. Therefore, as an attractive alternative to whole genome analysis, the transcriptome analysis can be used to explore the functional elements of the genome and reveal the expression mechanism of cells and tissues, especially for non-model organisms [5]–[6].

In the present study, we performed *de novo* transcriptome sequencing for *Salvia splendens* using the Illumina GA IIx sequencing platform. A total of 49,310 unigenes were identified, among which 134 differently expressed unigenes between Strain 35 and Cailinghong were mapped to 79 pathways. Moreover, we determined 2,453 simple sequence repeats (SSRs). This dataset was the first *Salvia splendens* transcriptomic data generated from massively parallel sequencing through *de novo* assembly. Our data expanded the repertoire of expressed sequences available for further genetic studies on this species.

## Materials and Methods

### Ethics statement

All necessary permits for field studies were obtained. The authority responsible for *Salvia splendens* farm is Beijing University of Agriculture, which provides permissions to collect the samples for our scientific research.

### Plant materials and RNA extraction

Branching traits between these two varieties show differences after stem has four nodes, so, we take samples when the forth node just emerge. Tissues including leaf, stem, shoot and root, were dissected from *Salvia splendens* of ten plants, and collected samples were then immediately frozen and stored in liquid nitrogen prior to further analysis. Total RNA was extracted from these materials using Norgen RNA Purification Kit (Norgen Biotek Co., Ontario, Canada). The quality and quantity of purified RNA were examined using an UltrasecTM 2100 pro UV/Visible Spectrophotometer (Amersham Biosciences, Uppsala, Sweden) and gel electrophoresis. Equal amounts of high-quality RNA from each material were pooled for cDNA synthesis.

### mRNA-seq library construction for Illumina sequencing

The mRNA-seq library was constructed using the mRNA-Seq Sample Preparation Kit (Cat. # RS-930-1001, Illumina Inc., San Diego, CA, USA) (Illumina) according to the manufacturer's instructions. Briefly, the poly-(A) mRNA was purified from total RNA samples using Magnetic Oligo (dT) Beads. To avoid the priming bias, the mRNA was fragmented by the RNA fragmentation kit (Ambion, Austin, TX, USA) before the cDNA synthesis. Cleaved RNA fragments were reversely transcribed into first-strand cDNA using reverse transcriptase (Invitrogen, Carlsbad, CA, USA) and random hexamer-primers. Subsequently, second-strand cDNA synthesis was carried out using DNA polymerase I (New England BioLabs, Ipswich, MA, USA) and RNaseH (Invitrogen, USA). The double-stranded cDNA was then end-repaired using T4 DNA polymerase (NEB), Klenow fragment (NEB) and T4 polynucleotide kinase (NEB). A single ‘A’ base addition using Klenow 3′ to 5′ exo-polymerase (NEB) was followed for the ligation of adapters, which have a single ‘T’ base overhang at their 3′ ends. Finally, modified cDNA was then ligated with PE Adapter Oligo Mix supplied by mRNA-Seq Sample Preparation Kit (Illumina) using T4 DNA ligase and incubated at room temperature for 15 min. The ligation products were purified using the MinElute PCR Purification Kit (QIAGEN, Dusseldorf, Germany) according to the manufacturer's instructions and then eluted with 10 µl of QIAGEN EB buffer. To select a size range of templates for downstream enrichment, the adaptor-ligated fragments were separated on an agarose gel through electrophoresis. cDNA fragments of the desired size range (200±25 bp) were excised and retrieved using a Gel Extraction Kit (Axygen Biosciences, Central Avenue Union City, CA, USA). To selectively enrich and amplify the cDNA fragments, PCR was performed using Phusion Master Mix (NEB) with two primers, PCR Primer PE 1.0 and PCR Primer PE 2.0 supplied by mRNA-Seq Sample Preparation Kit (Illumina). Briefly, after a denaturing step at 98°C for 30 sec, the amplification was carried out with 15 cycles at a melting temperature of 98°C for 10 sec, an annealing temperature of 65°C for 30 sec, and an extension temperature of 72°C for 30 sec. Finally, an extra extension step at 72°C for 5 min was performed, and then the temperature was maintained at 4°C. The amplified PCR products were purified using the QIAquick PCR Purification Kit (QIAGEN) according to the manufacturer's instructions and then eluted with 30 µl of QIAGEN EB buffer. After the adapter ligation and agarose gel separation, fractions of 150–200 bp were selected for library preparation. DNA concentration was determined through the quality control analysis, and the library was then validated using an Eppendorf Mastercycler ep realplex Real-Time PCR System. Subsequently, the mRNA-seq libraries were sequenced using a paired-end-read protocol with 2×100 bp of data collected per run on the Illumina Genome Analyzer IIx sequencing platform. Data analysis and base calling were performed by the Illumina instrument software.

### Sequence data analysis and assembly

Adapter sequences, low-quality sequences (reads with ambiguous bases ‘N’) and reads with more than 10% Q20 bases were all removed from the raw data. All sequences smaller than 60 bases were eliminated based on the assumption that small reads may represent sequencing artifacts [7]. The remaining reads were assembled into unigenes with Trinity program recovering more full-length transcripts across a broad range of expression levels, with sensitivity similar to methods that rely on genome alignments [8]. The overlap settings used for this assembly were 31 bp and 80% similarity, while all other parameters were set to their default values. To facilitate the access and utilization of the *Salvia splendens* transcriptome sequencing data, all the data, including the unigene sequences, annotations and relatively highly expressed genes, were uploaded to the ftp site (ftp.biomarker.com.cn) and a web site (http://lifecenter.sgst.cn/main/cn/salvia_unigene.jsp).

### Sequence annotation

The optimal assembly results were selected based on the assembly evaluation. A unigene database consisting of potential alternative splicing transcripts was obtained through the clustering analysis. SSR analysis of the unigenes longer than 1 kb was performed using the SSRIT software [9].

The assembled sequences were searched against the NCBI Nr and Nt databases (Last update was on March 1st, 2011) and Swiss-Prot database using BLASTn (version 2.2.14) with an E-value of 10^−5^. Each assembled sequence was given a gene name based on the best BLAST hit (highest score). Such search was limited to the first 10 significant hits for each query in order to increase the computational speed. The “getorf” program of EMBOSS software package [10] was used to predict the open reading frames (ORFs), with the longest ORF extracted for each unigene. Transcript levels were quantified in reads per kilobase of exon model per million mapped reads (RPKM) [11]. The RPKM measure of read density reflected the molar concentration of a transcript in the starting sample by normalizing for RNA length and the total read number in the measurement. Highly expressed genes were screened and listed.

The Swiss-Prot BLAST results were imported into Blast2GO [12], [13] in order to annotate the assembled sequences with GO terms describing biological processes, molecular functions and cellular components. These GO terms were assigned to query sequences, producing a broad overview of groups of genes catalogued in the transcriptome for each of three ontology vocabularies (biological process, molecular function and cellular component). ANNEX [14] was a tool used to enrich and refine the obtained annotation. The data presented herein represented a GO analysis at level 2, illustrating general functional categories.

The unigene sequences were also aligned to the COG database to predict and classify functions. KEGG pathways were assigned to the assembled sequences using the online KEGG Automatic Annotation Server (KAAS), http://www.genome.jp/kegg/kaas/. KEGG Orthology (KO) assignment was obtained using the bi-directional best hit (BBH) method [15]. The output of KEGG analysis consisted of KO assignments and KEGG pathways, which are populated with the KO assignments.

### Detection of differentially expressed unigenes

Differentially expressed unigenes between Strain 35 and Cailinghong were detected with IDEG 6 software [46]. General Chi squared test of statistical significance was used, and false discovery rate (FDR) of results were controlled. If FDR was lower than 0.01 and the highest RPKM of unigene was twice of the lowest one, this unigene was considered as differentially expressed unigene.

### EST-SSR detection

The obtained 49,310 unigenes of *Salvia splendens* were also subjected to the SSR detection using the online program: Simple Sequence Repeat Identification Tool (SSRIT, http://www.gramene.org/db/markers/ssrtool) [9], [16]. The parameters were adjusted for identification of perfect di-, tri-, tetra-, penta- and hexa-nucleotide motifs with a minimum of six, five, four, four and four repeats, respectively. Several information were included in this report as follows: the total number of SSR-containing sequences, sequence ID, SSR motifs, number of repeats (di-, tri-, tetra-, penta- and hexanucleotide repeat units), repeat length, SSR starts and SSR ends [16]. Moreover, mononucleotide repeats were ignored accordingly since it was difficult to distinguish the genuine mononucleotide repeats from polyadenylation products and single nucleotide stretch errors generated by sequencing.

## Results and Discussion

### Paired-end sequence analysis and de novo assembly

By using the Illumina Genome Analyzer, we generated about 100 bp independent reads from either end of a cDNA fragment. A total of more than 2 G bp reads were obtained from mRNA-seq whole transcriptome sequencing of both Strain 35 and Cailinghong. GC content of two samples was approximately 50%. Average Phred score value beyond 99% of the Cycle was greater than 20. These reads were considered as high-quality data for further analysis after above-mentioned stringent assessment and filtering. Table 1 shows an overview of the sequencing.

**Table 1:** Summary of transcriptome sequencing for Sal*via splendens*.

Sample	Read length	No. of Reads	Data(bp)	GC%	CycleQ20%
**Strain 35**	98.5+98.5	16,488,829	3,248,299,313	50.61	100
**Cailinghong**	91+91	16,464,713	2,996, 577,766	49.93	99.45055

Using the Trinity program, we assembled the obtained short-read sequences into 83,093 transcripts for Strain 35 with a mean length of 905 bp and into 81,127 transcripts for Cailinghong with a mean length of 919 bp. A N50 value of both assemblies was 1,346 bp. We found that 28,197 and 27,992 transcripts were longer than 1 kb in Strain 35 and Cailinghong, respectively. These transcripts were further clustered, resulting in 38,498 and 34,302 unigenes, among which 10,368 (26.94%) and 9,933 (28.95%) genes were greater than 1 kb in Strain 35 and Cailinghong, respectively. After blasting and clustering the unigenes of Strain 35 and Cailinghong, 49,310 unigenes (N50 value = 1,304 bp) for this species were obtained (Table S1). Table 2 and Table 3 exhibit an overview of the assembled transcripts and unigenes. These results demonstrated that the Illumina pyrosequencing possessed a potential of rapidly capturing a large number of transcriptomes.

**Table 2:** Summary of transcripts for Sal*via splendens*.

	Total length (percentage)	
Transcripts (bp)	Strain 35	Cailinghong
**200–300**	16,539(19.90%)	15,017(18.51%)
**300–500**	16,820(20.24%)	16,305(20.10%)
**500–1000**	21,537(25.92%)	21,813(26.89%)
**1000–2000**	20,903(25.16%)	20,882(25.74%)
**2000+**	7,294(8.78%)	7,110(8.76%)
**Total length**	75,182,756	74,543,320
**Count**	83,093	81,127
**N50 length**	1,346	1,346
**Mean length**	904.8025225	918.8472395

**Table 3:** Summary of unigenes for Sal*via splendens*.

	Total number (percentage)		
Unigene length (bp)	Strain 35	Cailinghong	*Salvia splendens*
**200–300**	11,392(29.6%)	9,481(27.6%)	15,208(30.8%)
**300–500**	9,003(23.4%)	7,643(22.3%)	11,839(24.0%)
**500–1000**	7,735(20.1%)	7,245(21.1%)	9,477(19.2%)
**1000–2000**	7,598(19.7%)	7,332(21.4%)	9,057(18.8%)
**2000+**	2,770(7.2%)	2,601(7.6%)	3,729(7.6%)
**Total length**	29,996,510	27,848,362	38,095,294
**Count**	38,498	34,302	49,310
**N50 length**	1,283	1,304	1,304
**Mean length**	779.1706063	811.858259	772.5673089

The obtained unigenes exhibited different lengths, ranging from 202 bp to 11.168 kb. A total of 8,471,368 (51.41%) reads were used for the assembly, of which 7,593,569 reads were uniquely against unigenes and 877,799 reads belonged to multi-position ones against unigenes. The percentage of reads used in the generation of unigenes was lower compared with those assembled (Solexa reads) in sweet potato root transcriptome [17], *coral larval* [18] and *Artemisia annua* [19]. This might be caused by the different platforms and the presence of alternative splicing regions [20] or repeats [21] in transcripts.

### Similarity analysis

**Figure 1:**
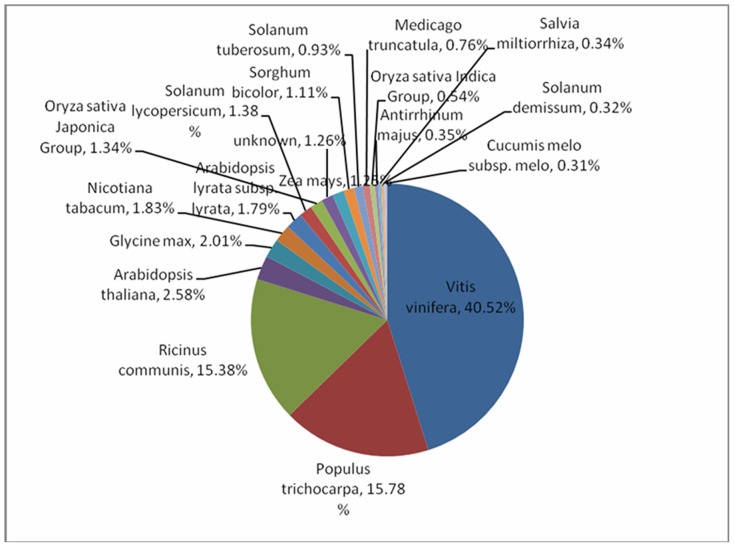
Similarity analysis based on the best hit.

All unigenes for *Salvia Splendens* were subjected to the BLASTx similarity analysis against the non-redundant (Nr) NCBI database. Among these unigenes, 33,925 (69%) had significant matches, and the remaining 15,385 (31%) demonstrated no significant hits. The identification of un-characterized sequences from cDNA libraries ranges considerably from 35% to 50% [22]–[24]. Based on the BLASTx similarity analysis of the unigenes, organism distribution showed that the unigenes hit a range of plant species. Among the various plants with protein sequences in Genbank, *Salvia splendens* unigenes had the highest number of hits to *Vitis vinifera* (40.52%), followed by *Populus trichocarpa* (15.78%), *Ricinus communis* (15.38%), *Arabidopsis thaliana* (2.58%) and *Glycine max* (2.01%). Figure 1 shows that the hit to *Salvia miltiorrhiza* was only 0.34%, and this was probably because of the insufficient sequences in Genbank. The high similarity of *Salvia splendens* unigenes to *Vitis vinifera* genes suggested the possibility of using *Vitis vinifera*'s ESTs as a reference sequence. These results also demonstrated the necessity of generating a large collection of *Salvia splendens* unigenes.

### Sequence annotation

Besides the NCBI Nr database, *Salvia splendens* unigenes were also aligned with several protein databases, Swiss-Prot, Kyoto Encyclopedia of Genes and Genomes (KEGG), Cluster of Orthologous Groups of proteins (COG), Gene Ontology (GO) and TrEMBL. Table 4 shows the overall functional annotation. Among the 49,310 unigenes, 24,888 (50.47%) had significant matches in the GO database, 23,167 (46.98%) had significant matches in the Nt database, while 25,371 (51.45%) had similarity to proteins in the Swiss-Prot database. Consequently, a total of 34,787 (70.55%) unigenes were successfully annotated in the Nr, Nt, Swiss-Prot, KEGG, COG, GO and TrEMBL databases (Table S2). The significance of the BLAST comparison partially depends on the length of the query sequence. Short reads obtained from sequencing would rarely be matched to known genes [3]. The percentage (29.45%) of unmapped unigenes in our study was relatively comparable to the percentage (30.84%) of short unigenes (200–300 bp). In other words, the short sequence reads generated by the sequencing technology and the corresponding short sequences of the assembly unigenes might mainly result in the low significance [4].

**Table 4:** Annotation of Unigene.

#Anno_Database	Annotated_Number	length≥300	length≥1000
COG_Annotation	9,896	3,334	5,572
GO_Annotation	24,888	10,445	10,363
KEGG_Annotation	6,995	2,631	3,299
Swissprot_Annotation	25,371	10,498	11,015
TrEMBL_Annotation	34,081	15,329	12,528
nr_Annotation	33,925	15,260	12,529
nt_Annotation	23,167	8,997	11,174
All_Annotated	34,787	15,645	12,555

### GO annotation

GO database is a collection of controlled vocabularies describing the biology of a gene product in any organism. There are three independent sets of ontologies: molecular function, biological process and cellular component [25]. Based on the annotation against the Nr Genbank database, a total of 167,388 GO terms were assigned to all 24,888 mapped unigenes, and averagely one unigene was assigned to seven GO terms. The majority of the GO terms were assigned to biological process (91,434, 54.62%), the molecular function (38,920, 23.25%) was in the middle, and the cellular component (37,034, 22.12%) was the least.

**Figure 2:**
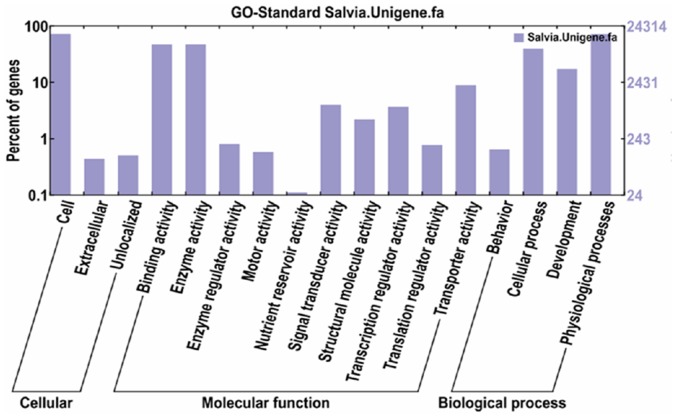
GO annotation.

Regarding the cellular component ontology, proteins involved in cell was dominant, among which the plasma membrane (GO: 0005886) was the most representative category. Under molecular function ontology, proteins for binding and enzyme activity were highly encoded by *Salvia splendens* unigenes. Moreover, biological process ontology contains mainly proteins involved in cellular processes and physiological processes, of which the oxidation reduction process (GO: 0055114) was the most representative GO term, followed by protein phosphorylation (GO: 0006468) (Figure 2). This distribution pattern indicated that *Salvia splendens* underwent multiple developmental processes [26].

### COG annotation

**Figure 3:**
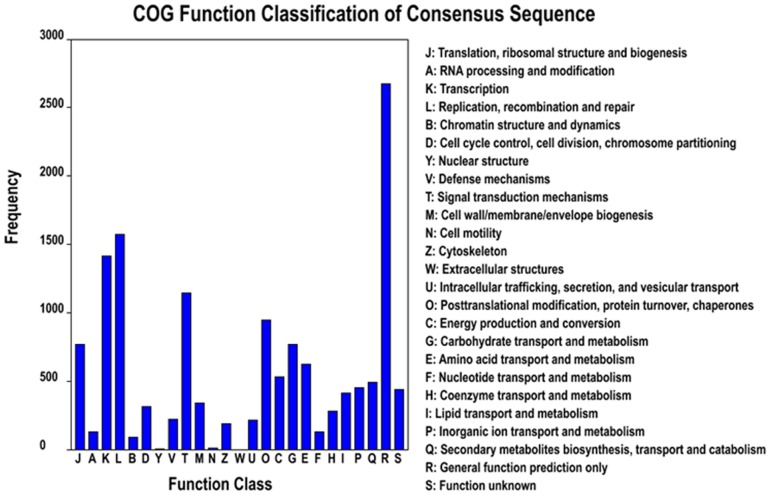
COG annotation.

As a useful platform for functional annotation of newly sequenced genomes, COG database classifies putative proteins into at least 25 protein families involved in cellular structure, biochemistry metabolism, molecular processing, signal transduction and so on (Figure 3). A total of 9,896 unigenes could be assigned to the COG classification according to the Nr database. The largest group was the cluster for general function prediction (2,612, 26.39%), followed by replication, recombination and repair (1,548, 15.64%), transcription (1,379, 13.93%), signal transduction mechanisms (1,138, 11.50%), posttranslational modification, protein turnover and chaperones (876, 8.85%), translation, ribosomal structure and biogenesis (752, 7.60%), as well as carbohydrate transport and metabolism (738; 7.46%). However, only six and 11 unigenes were assigned to nuclear structure and cell motility, respectively. In addition, no unigene was assigned to extracellular structures. These results exhibited that the growth and development of *Salvia splendens* was mainly based on the material and energy metabolism.

### Functional classification by KEGG

With the emphasis on biochemical pathways, KEGG pathway tool can be used as an alternative approach to categorize gene functions. A total of 6,995 unigenes were assigned to 259 biological pathways through this process. These predicted pathways were generally involved in the growth and development for compound biosynthesis, degradation, utilization, assimilation and pathways involved in the processes of detoxification and generation of precursor metabolites and energy. Enzymes encoded by annotated unigenes were grouped into almost all steps in several major plant metabolic pathways, including the Calvin cycle, gluconeogenesis, glycolysis, pentose phosphate pathway, several important secondary metabolite biosynthesis pathways and mitogen-activated protein kinase (MAPK) signaling pathway. This result suggested that a large number of metabolic activities occurred during the growth and development of *Salvia splendens*.

### Analysis of gene regulating shoot branching in Salvia splendens using the assembled unigenes

Meristems, defined by their determinacy, identity and position [27], control the organogenesis in plants [28]. The whole above-ground organs are derived from the shoot apical meristem (SAM), while the below-ground body of plants is generated by the root apical meristem. As the secondary meristem, axillary meristems, which form in the leaf axils, can give rise to branches or flowers. The Strain 35 is a different plant type from Cailinghong because of their various spatial and temporal sequences of axillary meristem initiation and outgrowth. Multiple factors can determine the formation and activity of axillary meristems, such as the genotype, developmental stage and environment. The integration of these multiple factors is likely to be mediated by a hormonal signaling network. A great deal of studies revealed complex interactions among the plant hormones in regulating shoot branching, including auxin, cytokinin (CK) and strigolactones (SL) [29], [30].

Auxin is mainly synthesized in the shoot apex, and then it is transported basipetally (downwards from the tip to the base) in the polar auxin transport stream (PATS). Depending on PATS, the growing shoot apex inhibits the outgrowth of axillary buds in the phenomenon termed ‘apical dominance’. Auxin can down-regulate the CK synthesis. CK and SL are synthesized in the root and acropetally transported (upwards from the base to the tip). CK regulates the meristem size, and its acropetal movement promotes the axillary bud outgrowth, while SL travels from the root to suppress the bud outgrowth.

We analyzed all unigene annotation (Table S2) and 134 different expressed genes. Some genes regulating the initiation and outgrowth of axillary meristem were also screened in our study. For example, *PIN1* family encodes auxin efflux carrier (17 homologous unigenes for Strain 35 and 11 homologous unigenes for Cailinghong), while *PID* family (4 homologous unigenes for Strain 35 and 3 homologous unigenes for Cailinghong) encodes a Ser/Thr protein kinase that phosphorylates and regulates the localization of PIN1. *YUC* genes (5 homologous unigenes for Strain 35 and 10 homologous unigenes for Cailinghong) of flavin monooxygenases are involved in local auxin biosynthesis. BARREN STALK1 (BA1) and LAX PANICLE (LAX) (9 homologous unigenes for Strain 35 and 5 homologous unigenes for Cailinghong) are two nuclear-localized basic helix-loop-helix putative transcription factors in *maize* and *rice*, respectively. They play roles in auxin-mediated initiation and outgrowth of axillary meristem.

SL is synthesized from the carotenoid pathway [31]. To date, several genetic and physiological models of branching control have been widely accepted, including carotenoid cleavage dioxygenase (CCD) enzymes (CCD7 and CCD8) (2 homologous unigenes for Strain 35 and 0 homologous unigenes for Cailinghong), cytochrome P450 monooxygenase (380 homologous unigenes for Strain 35 and 310 homologous unigenes for Cailinghong), F box and α/β-fold hydrolase [32]–[34]. Besides the hypothesis of auxin transport canalization, some other transcription factors can also regulate the axillary meristem function, such as the GRAS-type transcription factor (15 homologous unigenes for Strain 35 and 9 homologous unigenes for Cailinghong), HD ZIP class III transcription factor, NAC transcription factor and MYB transcription factor (79 homologous unigenes for Strain 35 and 73 homologous unigenes for Cailinghong).

Based on the sequence annotation, we showed that the number of unigenes related to auxin transport (including PIN and PID family) in Strain 35 was 21, greater than that (14) in Cailinghong. Polarity specification of adaxial/abaxial axis [GO: 0009944] are different (68 homologous unigenes for Strain 35 and 52 homologous unigenes for Cailinghong). These results were consistent with the apical dominance in Strain 35.

### SSR Discovery

As highly informative markers, SSRs have become one of the most widely used molecular marker systems for genetics, evolution and breeding studies. Previous study showed that putative SSR motifs can be detected from roughly 3–7% of expressed genes, mainly within the un-translated regions of the mRNA [35].

SSRs may have different putative functions. For example, gene expression can be manipulated by SSR variations in 5′-untranslated regions (UTRs) through affecting the transcription and translation; transcription slippage is induced by SSR expansions in the 3′-UTRs, resulting in expanded mRNA; and intronic SSRs can affect the gene transcription, mRNA splicing, or export to cytoplasm. Therefore, SSRs within genes should be subjected to stronger selective pressure compared with other genomic regions [36]. To investigate SSR profiles in the unigenes of *Salvia splendens*, a total of 49,310 unigene sequences were submitted to an online tool for SSR discovery. As a result, 2,453 SSRs were obtained from these unigenes (4.9%). Table 5 shows that among these SSRs, di-nucleotide repeat motif was the most abundant, accounting for 979/2453 (39.9%), followed by tri-nucleotide repeat motif (719/2453, 29.3%), tetranucleotide (20/2453, 0.8%), penta-nucleotide (17/2453, 0.7%) and hexa-nucleotide (17/2453, 0.7%) repeat units.

**Table 5:** EST-SSRs of *Salvia splendens*.

SSR Type	SSR Number
perfect_SSR_1	566
perfect_SSR_2	979
perfect_SSR_3	719
perfect_SSR_4	20
perfect_SSR_5	17
perfect_SSR_6	17
compound_SSR (eg.ATATGCGC)	130
compound_SSR* (eg. AAAAAGAG)	5
Total_SSR	2,453

We showed that the AG/GA/CT/TC motifs constituted approximately half of the total number of di-nucleotide SSRs (Table 6), and similar finding has been reported in *Huperzia serrata Thunb* [38]. CT repeats were the most commonly detected motif among the di-nucleotide repeat motifs. This result was different from that of *H. serrate* or *Arabidopsis*, in which AG repeats are the most frequent. This might be caused by the introduction of additional repeats during the chromosome replication [39]. Since the same motif (TCTCTCTCT) is detected in a 60-nt region downstream of the transcription start site of CaMV 35S RNA, (CT)_n_ may function as an enhancer to manipulate the gene translation in plant protoplasts [40]. Furthermore, (GA)_n_ possesses complementary sequences to (CT)_n_, and it functions as regulatory elements containing a series of overlapped GAG motifs (AGAGAGa) involved in light regulation [41], [42]. Our findings were coincident with those of *Arabidopsis*, *rice* and *Moso bamboo* when comparing the frequency of di- or tri-nucleotide motif of SSRs among the unigenes of *Salvia splendens*, in which the type and distribution of tri-nucleotide SSRs are also the most abundant [37]. Similar to those of *Moso bamboo* and *rice*, AAG/AGC/CTC/GGA/TCT/CTG/GAA/TTC (30.83% of tri-nucleotides) were the most commonly detected motif for tri-nucleotide repeats of SSRs (Table 6). This could be correlated with the higher G+C content of herbaceous plants, leading to more frequent insertion/deletion of certain nucleotides, without causing frame shift mutations [43].

**Table 6:** Repeats of di- and tri-nucleotides.

repeats	Number of repeats								total
	5	6	7	8	9	10	11	12	
AC		5		1	3	6			15
AG		52	17	9	17	7	6		108
AT		9	5	5	2		1		22
CA		7	5	1	4	1	2		20
CT		34	27	11	13	17	6	1	109
GA		31	21	19	9	18	7		105
GC			1						1
GT		4	3	4	3		4		18
TA		7	7		2	1			17
TC		29	14	20	7	12	5		87
TG		10	6	6	3	5	1		31
AAC	3								3
AAG	12	4	2	1					19
AAT	1		1						2
ACA	1	1							2
ACC	2								2
ACG	1								1
ACT		1							1
AGA	9	2	1						12
AGC	8	6	1						15
AGG	2	1							3
AGT	1	1		1					3
ATC	4	2							6
ATG	4								4
ATT	4	1							5
CAA	1								1
CAC	2	1	1						4
CAG	4	3							7
CAT	10		1	1					12
CCA	5	3	2						10
CCG	6	2							8
CCT	1	2							3
CGC	7	1							8
CGG	7	4	1						12
CGT	1								1
CTA	1								1
CTC	8	5	1						14
CTG	8	5							13
CTT	4	4							8
GAA	4	5	3	1					13
GAC	2								2
GAG	12								12
GAT	7	3	1						11
GCA	1	3							4
GCC	3	4							7
GCG	7	2	1						10
GCT	5		1						6
GGA	11	2	1						14
GGC	4	2							6
GGT	6	1	3						10
GTC	1								1
GTG	7								7
GTT	2	1							3
TAC		2							2
TAG	3	3							6
TCA	6	2	2						10
TCC	4	3	1						8
TCG	1								1
TCT	8	6							14
TGA	5		3						8
TGC	5	1	2						8
TGG	4	2	3	1					10
TGT	3								3
TTA		1							1
TTC	7	3	3						13
TTG	3								3
total	238	283	141	81	63	67	32	1	906

Since SSRs are ubiquitous in transcriptomes, typically locus-specific and codominant, multi-allelic, highly polymorphic and transportable among species within genera, they have been developed as powerful molecular markers for comparative genetic mapping and genotyping [44], [45]. As a rich source of SSRs, EST databases can be used for genotyping in numerous species of flowering plants. The unigenes from *Salvia splendens* obtained in our study provided a good resource for SSR mining and applications in research and molecular marker-assisted breeding.

### Conclusions

In the present study, we, for the first time, performed *de novo* transcriptome sequencing analysis of *Salvia splendens* tissues using the Illumina platform. To our knowledge, this was the first investigation on the whole transcriptome of *Salvia splendens* using the Illumina paired-end sequencing technology, and the reads were assembled without a reference genome. More than 2.2 G bp of data were generated and assembled into 49,310 unigenes. Furthermore, we identified a large number of candidate genes potentially involved in growth, development, flowering and plant hormone pathways. In addition, a large number of SSRs were detected. This dataset might provide useful information about the molecular mechanisms of branching and other biochemical processes in *Salvia splendens*.

## Supporting Information

Table S1 **49310 unigenes for** ***Salvia splendens*** **(FA).**
(FA) Click here for additional data file.

Table S2 **The annotation by NCBI Nr, Nt, Swiss-Prot, KEGG, TrEMBL, COG and GO seqdb databases (XLS).**
(XLS)
